# Avenanthramide supplementation attenuates exercise-induced inflammation in postmenopausal women

**DOI:** 10.1186/1475-2891-13-21

**Published:** 2014-03-19

**Authors:** Ryan Koenig, Jonathan R Dickman, Chounghun Kang, Tianou Zhang, Yi-Fang Chu, Li Li Ji

**Affiliations:** 1 Department of Kinesiology, University of Wisconsin–Madison, Madison, WI 53706, USA; 2 Laboratory of Physiological Hygiene and Exercise Science, University of Minnesota, 1900 University Avenue, Minneapolis, MN 55455, USA; 3 Quaker Oats Center of Excellence, PepsiCo Nutrition, 617 W Main Street, Barrington, IL 60010, USA

## Abstract

During aging, chronic systemic inflammation increases in prevalence and antioxidant balance shifts in favor of oxidant generation. Avenanthramide (AVA) is a group of oat phenolics that have shown anti-inflammatory and antioxidant capability. The present study investigated whether dietary supplementation of avenanthramides (AVA) in oats would increase antioxidant protection and reduce inflammation after a bout of downhill walking (DW) in postmenopausal women. Women at age of 50–80 years (N = 16) were randomly divided into two groups in a double-blinded fashion, receiving two cookies made of oat flour providing 9.2 mg AVA or 0.4 mg AVA (control, C) each day for 8 weeks. Before and after the dietary regimen, each group of subjects walked downhill on a treadmill (−9% grade) for 4 bouts of 15 minutes at a speed of 4.0 km/h with 5 minutes rest between sessions. Blood samples were collected at rest, 24 h post-DW, and 48 h post-DW pre- and post-supplementation. Both DW sessions increased plasma creatine kinase activity (P < 0.05). Before supplementation, in vitro neutrophil respiratory burst (NRB) activity was increased at 24 h post-DW (P < 0.05) and C-reactive protein (CRP) was increased 48 h post-DW (P < 0.05). AVA supplementation decreased DW-induced NRB at 24 h (P < 0.05) and CRP level 48 h (P < 0.05). Plasma interleukin (IL)-1β concentration and mononuclear cell nuclear factor (NF) κB binding were suppressed at rest and during post-DW period in AVA but not C group (P < 0.05). Plasma total antioxidant capacity (P < 0.05) and erythrocyte superoxide dismutase activity were increased in AVA vs. C (P < 0.05), whereas glutathione redox status was elevated 48 h post-DW but not affected by AVA. Thus, chronic AVA supplementation decreased systemic and DW-induced inflammation and increased blood-borne antioxidant defense in postmenopausal women.

## Introduction

The skeletal muscle of aged individuals decreases muscle mass, force generation and metabolic functions known as sarcopenia. Recent research points to a strong link between aging and inflammation [1,2]. So many diseases have been identified to have an etiological origin of inflammation that the term “inflammaging” has been coined [3-6]. Therefore, developing strategies to prevent and reduce inflammation in the aging population has become a priority in gerontological research in recent years.

The increase in inflammation during aging has been linked to increased nuclear factor (NF) κB binding to DNA in many organs and tissues, as well as several types of blood borne cells [7]. NFκB is sensitive to oxidative stress and a variety of other stimuli and is responsible for the regulation of the transcription of a variety of gene targets, including pro-inflammatory cytokines such as interleukin (IL)-1, 6 and tumor necrosis factor (TNF)-α [8]. Aged rats and mice displayed increased nuclear NFκB binding activity in several major organs studied, whereas no increase in cytoplasmic NFκB was observed [9]. IL-1 and 6 gene expression in the T cells of older human subjects was elevated compared with younger counterparts with or without NFκB induction, indicating aging is associated with immune dysregulation resulting in a pro-inflammatory state [10].

In women the phenomenon of menopause results in a lack of production of estrogen, which adds complexity to the aging milieu. Estrogens function as antioxidants, and their absence in postmenopausal women could contribute to an increased susceptibility to oxidative stress [11]. Estrogen was recently shown to up-regulate antioxidant enzymes via mitogen activated protein kinase (MAPK) and NFκB pathways [12]. In addition estrogens may function to stabilize cell membranes and to regulate cell signaling through the binding to estrogen receptors [13]. These mechanisms are thought to provide important protection from muscle damage to women following a bout of unaccustomed exercise. Indeed, postmenopausal women exhibited increased serum creatine kinase (CK) and lactate dehydrogenase (LDH) as well as increased mRNA expression of pro-inflammatory cytokines following strenuous eccentric exercise compared to their counterparts with hormone therapy [14].

Downhill walking is a muscular activity that involves lengthening or eccentric contraction (EC) and breaks weaken myofibrils and activate proteases and lipases, followed by immunological responses such as infiltration of neutrophils, free radical generation and expression of pro-inflammatory cytokines and chemokines [15]. NFκB activation escalates the process and provokes systemic inflammation that could have broad health outcomes such as muscle pain, chronic inflammation (rheumatoid), leading to underperformance and fear of participation in exercise and sports. However, recent research have shown pharmacological treatments of EC-induced inflammation such as NSAID might interrupt normal healing process and large doses supplementation of antioxidants of pharmaceutical source can be more detrimental than beneficial as it interferes with intrinsic adaptive responses and sometimes takes away the benefit of exercise [16,17]. Thus, seeking phytochemicals demonstrating antioxidant and anti-inflammatory properties for daily dietary supplements is desirable.

Oat (*Avena sativa*), although consumed in considerably lower quantities worldwide than wheat and rice, has a highly edible quality and contains high antioxidants such as tocopherols, tocotrienols, and flavonoids [18]. In addition, oat contains a unique group of approximately 40 different types of avenanthramides (AVA) that consist of an anthranilic acid derivative and a hydroxycinnamic acid derivative linked by a pseudo- peptide bond [19]. Of all the AVA that have been identified, three stand out due to their abundance and have been labeled as AVA-A, −B, and –C, which differ by a single moiety on the hydroxycinnamic acid ring. All three AVA of interest showed antioxidant activity with AVA-C being the most potent [20]. Additional studies performed have shown that AVA have the anti-inflammatory and anti-atherogenic effects of decreasing monocyte adhesion to human aortic endothelial cells (HAEC), as well as their expression of adhesion molecules and pro-inflammatory cytokines [21]. AVA-C displayed further antiatherogenic potential by inhibiting vascular smooth muscle cell (SMC) proliferation and enhancing nitric oxide production in both SMC and HAEC in parallel with the up-regulation of mRNA expression of endothelial nitric oxide synthase [22]. These effects were shown to be derived from decreased NFκB activity [23].

The antioxidant, anti-inflammatory, and NFκB inhibitory properties of AVA make it a candidate for supplementation in the cause of decreasing inflammation and muscle damage in post-menopausal women. Thus, the present study was designed to test the anti-inflammatory and antioxidant capability of AVA in postmenopausal women. We hypothesize that AVA supplementation would increase plasma antioxidant defense, inhibit NFκB-DNA binding in the mononuclear cells and decrease DW-induced systemic inflammation.

## Materials and methods

### A. Subjects

Older women aged 50–80 years were recruited from the Madison, Wisconsin, community or from faculty and staff of the University of Wisconsin-Madison. The recruitment procedure and study conducts were approved by the Health Science Institutional Review Board for Human Subjects of UW-Madison. The subjects were randomly assigned to one of two groups (N = 8 per group) receiving a high dose of AVA supplementation in the diet, or receiving a low dose of AVA present in normal oats, serving as Control. Other than this difference, the groups were treated exactly the same in a double-blind fashion.

All participants gave informed consent before enrolling in the study. They also completed a Health History Survey to ensure that they were eligible for the study and healthy enough to exercise. Criteria for exclusion from the study were smoking or other tobacco use, drinking alcohol in excess of 5 drinks per week, use of dietary antioxidants, blood pressure medication, non-steroidal anti-inflammatory drugs (NSAIDs) and anticoagulants or antidiabetic or hypoglycemic drugs.

### B. Dietary supplementation

Because the goal of the study is to study the biological efficacy of AVA, we employed a dietary regimen wherein both groups of subjects were supplemented with oats, which differed only in AVA concentration but processed identical nutritional and antioxidant contents (see below). Both dietary groups of subjects received cookies made with oat samples with standardized AVA concentration provided courtesy of Ceapro Inc. (Edmonton, AB, Canada). AVA concentrations were verified by high-performance liquid chromatography (HPLC) in our laboratory. The high-AVA oat flour contained 190 mg/kg, and the Control group flour contained 8 mg/kg, the lowest AVA concentration among all oat lines available. The recipe for each type of cookie was identical except in the type of flour used. Each cookie contained 30 g flour (high- or low-AVA), 5.91 mL unsweetened apple sauce (Surefine), 12.32 mL artificial sweetener (Natrataste Gold), 0.616 mL baking soda, and 0.0308 mL table salt. They were baked in a low temperature oven (121°C) for 15 minutes to ensure that AVA was not broken down or produced by the oat during the process. AVA concentration in the high-AVA cookies was 4.6 mg/cookie (9.2 mg/day), and it was 0.2 mg/cookie (0.4 mg/day) in the control cookies. Each cookie provided 125 kilocalories (250 kcal/day) regardless of group.

Supplementation began on the evening of the second study visit following the third blood draw and ended on the evening of the third study visit following the fifth blood draw (see below). Subjects were furnished with cookies and instructed to consume two per day: one in the morning with breakfast and one in the evening with dinner.

### C. Study visits

A total of six visits were required for each subject following recruitment and consenting. There were three pre-supplementation visits and three post-supplementation visits identical to the pre-supplementation visits. The first visit of each trio consisted of completion of the International Physical Activity Questionnaire (IPAQ) and health history questionnaire, downhill walking (DW), and a blood draw. The second and third visit occurred 24 hours after the first and consisted of a single blood draw. The pre-supplementation and post-supplementation visits were separated by 8 weeks of dietary supplementation regimen as described above.

### D. Downhill walking

DW was performed on a treadmill in the UW Biodynamics Laboratory. Each of the 2 DW sessions consisted of 4 bouts of 15 minutes of treadmill walking separated by 3 sessions of 5 minutes of quiet rest. The treadmill grade was set at −9% and the speed to 4.0 km/h. Heart rate was recorded every 5 minutes using a heart rate monitor.

### E. Blood sample collection and preparation

Mixed venous blood was drawn from an antecubital vein into 4 EDTA-coated Vacutainer tubes (7 mL each, Fisher Scientific). Whole blood was placed on ice and then immediately centrifuged at 500 × *g* at 4 degrees C for use in the glutathione assay (see below) or gently pipetted over two layers (3 ml of each) of density gradient (Histopaque and Ficoll-Paque) for isolation of blood cells. After centrifugation at 500 × *g* for 30 minutes at 20°C, plasma was removed by aspiration and frozen at −80°C. A band of monocytes was then removed by aspiration, washed with phosphate buffered saline (PBS), and frozen at −80°C. Next, the remaining fluid (not packed erythrocytes) was removed and washed with ice cold PBS to attain neutrophils. Any erythrocytes contaminating the sample were lysed with the addition of nanopure water. After gentle inversion, tonicity was restored by the addition of 3% NaCl. After centrifugation at 900 × *g* for 5 minutes at 4°C, the neutrophil pellet was resuspended in Hank’s balanced salt solution (HBSS) and the cells counted by microscope and hemacytometer and diluted to 1.5 × 10^6^ cells/mL for immediate analysis of respiratory burst (see below). Packed erythrocytes were removed and stored immediately at −80°C.

### F. Biological measurements

#### 1. ELISA

Enzyme-linked immunosorbent assay (ELISA) kits (eBioscience, Read-Set-Go! ELISA, San Diego, CA) were used to test for the plasma concentrations of interleukin (IL)-1β, IL-6, tumor necrosis factor (TNF)-α, and C-reactive protein (CRP) per manufacturer’s instruction. All samples were measu**r**ed in duplicate using 96-well plates coated with capture antibody. Following sample addition, detection antibody, avidin horse radish peroxidase, and enzyme substrate were added in succession with each step separated by room-temperature incubation and thorough washing with a PBS-Tween 20 wash buffer. Absorption at 525 nm was measured on a plate reader (Spectra MAx 340, Molecular Devices) and used to determine plasma concentration from a standard curve generated using recombinant standards provided by the manufacturer.

NFκB binding to DNA was measured by ELISA in nuclear extracts of mononuclear cells. The assay principle is as above; however, only p65 bound to DNA was detected. Nuclear extraction was conducted according to manufacturer’s instructions (Millipore Nuclear Extraction Kit). Manufacturer’s instructions were followed for the ELISA process, which utilized an antibody against p65 (eBioscience InstantOne ELISA). Samples were scanned using a luminometer (Turner Biosystems #2030-000).

#### 2. HPLC

##### **a. Plasma glutathione**

Glutathione concentrations were measured by HPLC based on the method described by Ji and Fu [24]. Both GSH and GSSG were detected, and the ratio of GSH:GSSG calculated. This assay was performed on plasma separated from a blood sample that was kept on ice and centrifuged at 4°C immediately upon being drawn. 250 μL of plasma was transferred to a tube containing 10 μL of 0.4 mmol/L iodoacetic acid and excess sodium bicarbonate. After incubation at room temperature for 1 hour, 2 μL of 2,4-dinitrofluorobenzene (Sanger’s reagent, Sigma Chemical, St. Louis, MO) was added, and the samples were kept in the dark for 28 hours before the HPLC detection. Concentrations of GSH and GSSG were determined using a Shimadzu UV–VIS detector at 365 nm wavelength and quantified with standard curves generated using GSH and GSSG standards.

##### **b. Avenanthramide concentration**

Cookie AVA concentrations were measured using HPLC. The method of Chen et al. [25] was modified for use on homogenized cookies. To 200 μl of sample, 20 μl of vitamin C-EDTA was added. Then 500 μl of 100% acetonitrile (ACN) was added to the tubes. After 5 minutes, the samples were centrifuged at 15000 × *g* for 5 min. The supernatant, which contained the AVA, was removed, and the solvent was evaporated by motorized vacuum pump (Fisher Scientific) at a pressure of approximately 200 mm Hg for approximately 5 minutes. The residue was reconstituted in 200 μl of HPLC aqueous solvent. Again the samples were centrifuged at 15000 × *g* for 5 min. The supernatant, which contained the AVA, was removed, and the solvent was evaporated by motorized vacuum pump (Fisher Scientific) at a pressure of approximately 200 mm Hg for approximately 5 minutes. The residue was reconstituted in 200 μL of HPLC aqueous solvent. Again the samples were centrifuged at 15000 × *g* for 5 min. All samples were analyzed for AVA concentration with a procedure based on Milbury [26] on a dual pump Shimadzu HPLC system with a UV–VIS spectrophotometric detector, a Supelco C18 column with inline guard column. Absorption at 330 nm was tracked by Shimadzu EZStart 7.2.1 software.

#### 3. Neutrophil respiratory burst (NRB)

Neutrophils diluted in HBSS to 1.5 × 10^6^ cells/mL were assayed for respiratory burst activity by luminometer using a procedure according to Benbarek et al. [27] with modifications. Neutrophils were incubated with luminol (Sigma, MO) for 5 minutes at 37°C in a shaking water bath. Three concentrations of phorbol myristate acetate (PMA) were used to evaluate the effects of maximal, moderate, and minimal stimulation of the cells. The highest concentration was 160 μmol/L, the middle concentration was 16 μmol/L, and the low concentration was 1.6 μmol/L. A total of 1 × 10^6^ neutrophils were used in each trial. The respiratory burst chemiluminescence was tracked for 30 minutes by luminometer (Turner Biosystems) with 10 measurements in one second of individual samples every 2.5 minutes. A cell-free blank containing equal volume of HBSS received luminol and maximal PMA concentration in order to measure chemiluminescence not associated with cellular activity. The mean of 10 measurements at each time point was calculated and the time course of the respiratory burst plotted. Area under curve was calculated by the trapezoidal rule and used as a measure of total respiratory burst activity.

#### 4. Spectrophotometric assays

##### a. Plasma total antioxidant capacity

Plasma total antioxidant capacity (TAC) was measured by spectrophotometer by monitoring the attenuation of 2,2’-azinobis-3-ethylbenzothiazoline-6-sulfonic acid (ABTS) oxidation at 734 nm according to Re et al. [28]. A solution of 7 mmol/L ABTS and 2.45 mmol/L aluminum potassium sulfate (APS) was made immediately before the conducting of the assay and kept in the dark. An aliquot of 100 μL plasma was added to a final volume of 1 mL with ABTS/APS solution. The cuvette was mixed by inversion and then incubated at 37°C for 5 minutes. The cuvettes were then read against a Trolox (6-hydroxy-2,5,7,8-tetramethylchroman-2-carboxylic acid) standard curve using a spectrophotometer (Shimadzu UV160).

##### b. Plasma creatine kinase

Plasma ceatine kinase (CK) activity was measured as a marker of eccentric muscle damage according to the procedure of Tanzer and Gilvarg [29]. The CK reaction was coupled to NADH conversion to NAD by lactate dehydrogenase (LDH), which, along with pyruvate kinase (PK), phosphoenol pyruvate (PEP), and NADH, were present in the reaction mixture. The decrease in NADH concentration was tracked using a spectrophotometer (Shimadzu UV160).

##### c. Erythrocyte superoxide dismutase

Erythrocyte superoxide dismutase (SOD) activity was measured by spectrophotometrically by tracking the decrease in auto-oxidation of epinephrine to adrenochrome according to Sun and Zigman [30]. Epinephrine autoxidation rate was measured at 320 nm for 3 minutes in the presence of an aliquot of erythrocyte lysate. The slope of the linear portion of the absorption graph was used to determine SOD activity by determining the percent inhibition of epinephrine autoxidation via comparison to the blank. Activity was normalized to hemoglobin concentration.

##### d. Erythrocyte glutathione peroxidase

Erythrocyte glutathione peroxidase (GPx) activity was measured by monitoring the change in NADPH concentration in a system with excess GSH and glutathione reductase (GR) in the presence of H_2_O_2_[24]. Activity was normalized to hemoglobin concentration.

Erythrocyte hemoglobin was measured using Drabkin’s reagent (potassium ferricyanide and potassium cyanide in sodium bicarbonate), which binds hemoglobin to cause a shift in maximal absorbance, which can be measured by the spectrophotometer [31].

### G. Statistical analysis

Data were shown as mean ± SEM and analyzed using the Planned Comparison method. A three-way repeated measures ANOVA was conducted using R (version 2.14.1) statistical software. The three main factors are (a) post- vs. pre-AVA supplementation, (b) timing with respect to DW test (rest vs. 24 h post-DW vs. 48 h post-DW), and (c) high-AVA vs. Control supplementation. The standard error of estimate of the ANOVA was used to complete a priori planned comparisons. Significance for each comparison was set at *P* < 0.00455, which is the quotient of 0.05 divided across the 11 comparisons.

## Results

### A. Participant data

The age, height, weight, and body mass index (BMI) of the study participants are displayed in Table 1. There were no significant differences between groups for any of the parameters measured. Body weight and BMI were unchanged following dietary supplementation.

**Table 1:** Characteristics of postmenopausal participants

			**Pre-Supplementation**		**Post-Supplementation**	
	Age (y)	Height (m)	Body Weight (kg)	BMI	Body Weight (kg)	BMI
Control	60.125 ± 2.20	1.48 ± 0.025	57.50 ± 2.42	26.40 ± 1.59	57.65 ± 2.53	26.42 ± 1.61
AVA	59.000 ± 2.25	1.45 ± 0.051	60.45 ± 2.66	29.01 ± 1.27	59.91 ± 2.69	28.74 ± 1.39

Note: Data are mean ± SEM. AVA, avananthramide. BMI, body mass index defined by the ratio of body weight (kg) divided by height (meter)^2^.

### B. Muscle damage caused by DW

**Figure 1:**
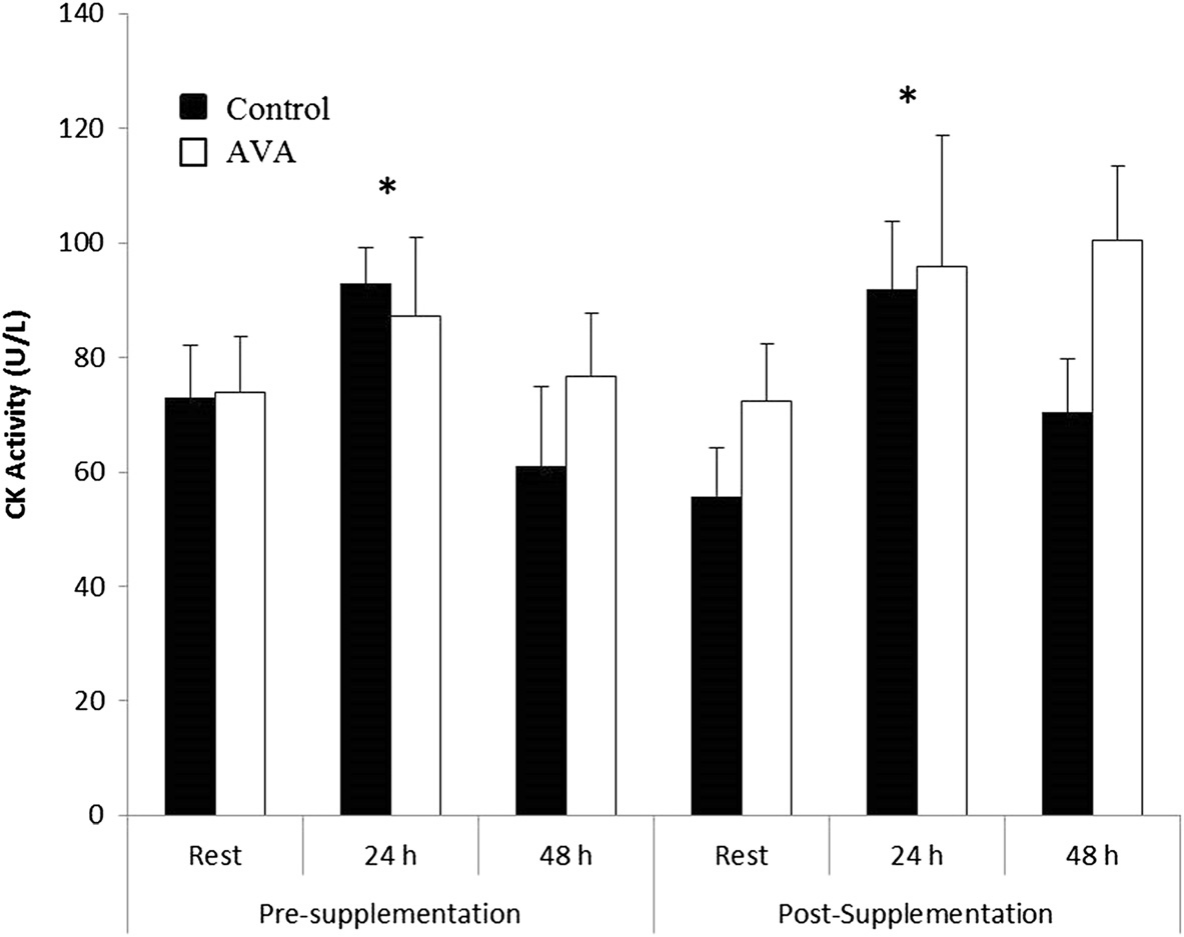
**Plasma CK activity in postmenopausal women in response to downhill walking (DW).** Data are mean ± SEM (N = 8). * *P* < 0.05, 24 h post-DW vs. Rest.

Plasma CK activity was significantly elevated 24 h after DW both before and after the dietary supplementation regimen (*P* < 0.05; Figure 1). CK activity was not different 48 h after DW compared to resting levels in both groups. No difference was observed between AVA and control across all groups.

### C. Inflammatory markers

**Figure 2:**
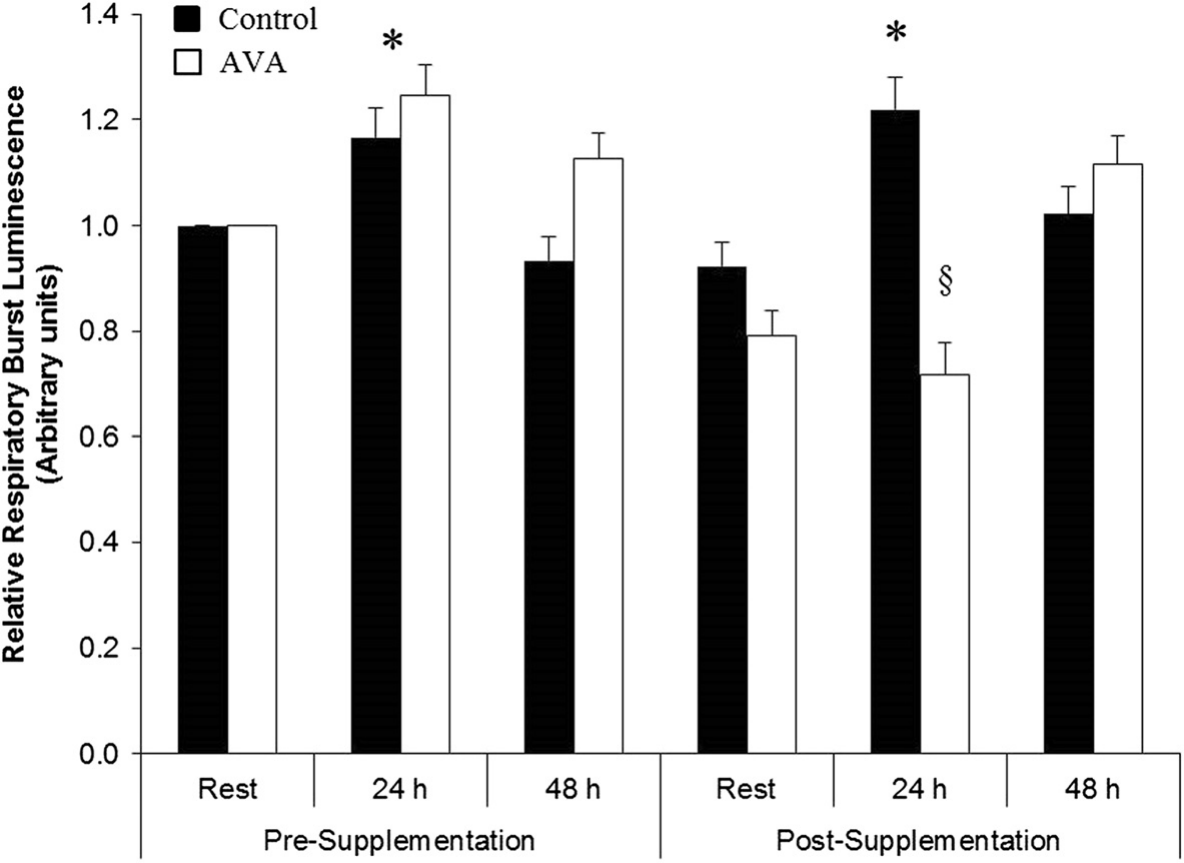
**Neutrophil respiratory burst activity in postmenopausal women in response to DW.** Data are mean ± SEM (N = 8), normalized to Pre-Supplementation Rest value. * *P* < 0.05, 24 h vs. Rest. § *P* < 0.05, AVA vs. Control in 24 h post-DW.

In the current study inflammatory responses to DW by older women subjects were assessed by several biomarkers including neutrophil respiratory burst (NRB) activity *in vitro*, plasma CRP concentration and pro-inflammatory cytokine levels. NRB activity increased significantly 24 h after DW before supplementation (*P* < 0.05; Figure 2). This post -DW elevation of NRB activity maintained in Control group after 8 week dietary regimen, but was abolished in the high-AVA supplemented group (*P* < 0.05).

**Figure 3:**
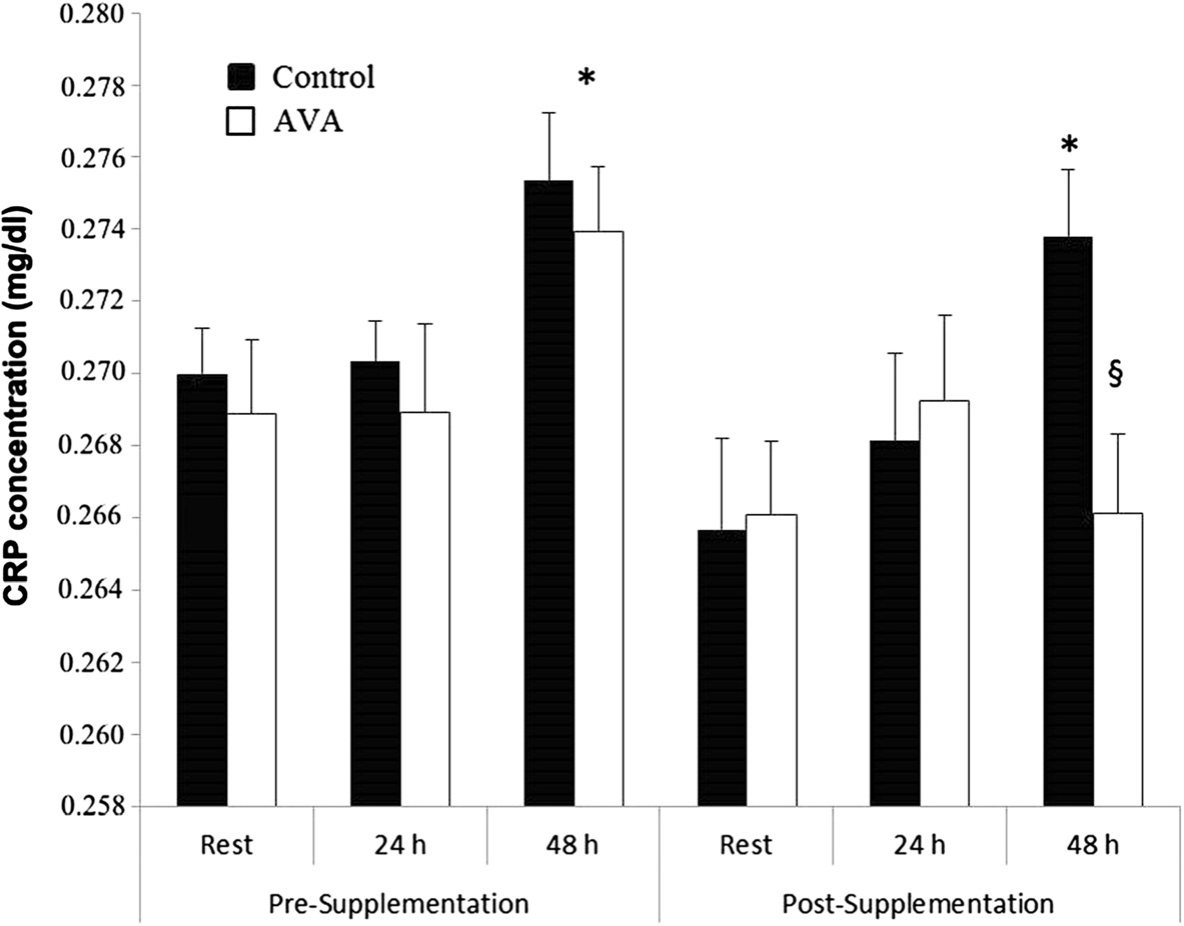
**Plasma C-reactive protein (CRP) concentrations in postmenopausal women in response to DW.** Data are mean ± SEM (N = 8). * *P* < 0.05, 48 h post-DW vs. Rest. § P < 0.05, AVA vs. Control in 48 h post-DW.

Plasma level of CRP was not different between rest and 24 h post-DW either before or after the 8 week supplementation, but increased significantly 48 h after DW prior to supplementation (*P* < 0.05; Figure 3). Following supplementation, plasma CRP level was elevated 48 h post-DW only in Control group but not in AVA group (*P* < 0.05).

**Figure 4:**
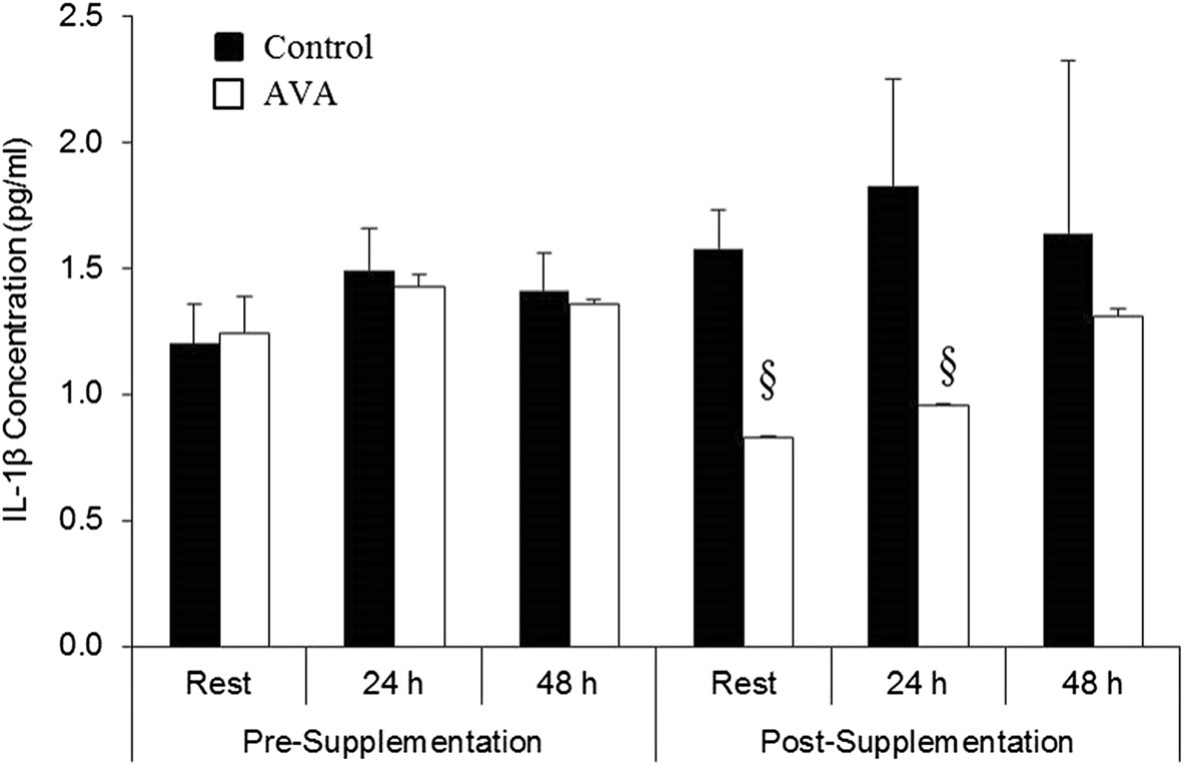
**Plasma interleukin (IL)-1β concentrations in young women in response to DW.** Data are mean ± SEM (N = 8). § *P* < 0.05, AVA vs. Control in Rest and 24 h post-DW.

Plasma IL-1β concentration was not altered by DW prior to dietary supplementation (Figure 4). Following 8 wk of high-AVA supplementation, IL-1β level at rest and 24 h post-DW was decreased by nearly 50% in the high-AVA group compared to their Control counterparts (*P* < 0.05). This difference vanished at 48 h post-DW.

We measured plasma concentration of two other pro-inflammatory cytokines, TNF-α and IL-6. TNF-α levels were not affected by DW before or after dietary supplementation regimen, but showed a strong trend to decrease (0.05 < P < 0.1) after supplementation (data not shown). IL-6 levels were not affected by DW or AVA supplementation (data not shown).

**Figure 5:**
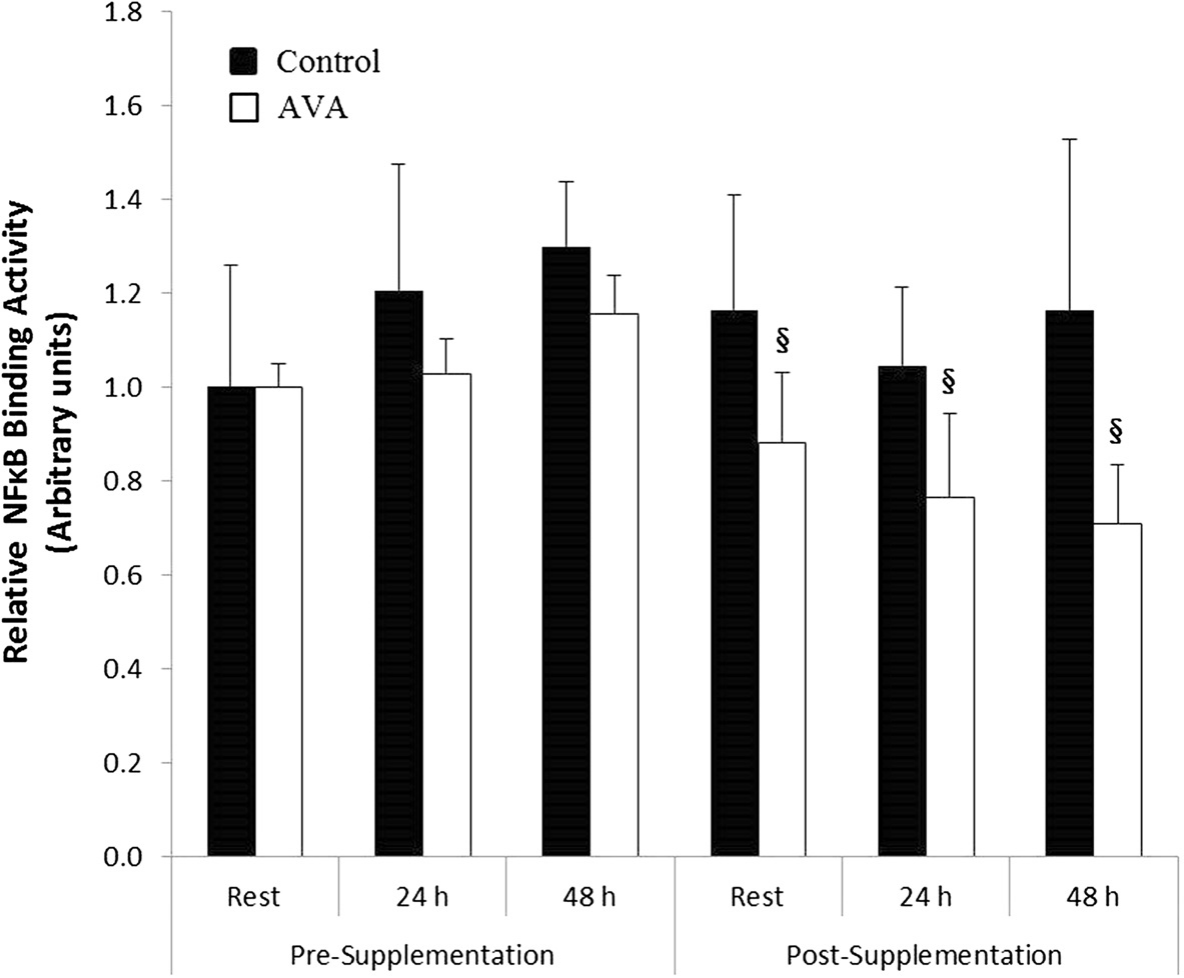
**Mononuclear cell NFκB binding activity in postmenopausal women in response to DW.** Data are mean ± SEM (N = 8), normalized to Pre-Supplementation Rest value. § *P* < 0.05, AVA vs. Control.

DW did not significantly affect monocyte NFκB binding activity before dietary supplementation (Figure 5). After supplementation NFκB binding was lower in AVA vs. Control at rest as well as 24 and 48 h post-DW (*P* < 0.05).

### D. Antioxidant defense

**Figure 6:**
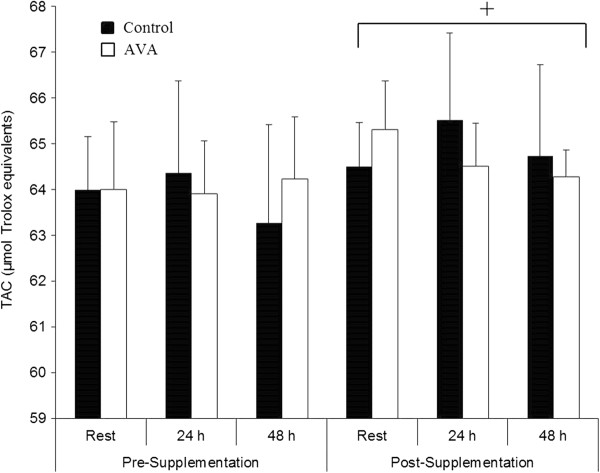
**Plasma total antioxidant capacity (TAC) in postmenopausal women.** Data are mean ± SEM (N = 8). + *P* < 0.05, Post- vs. Pre-supplementation regardless of time or AVA treatment.

Plasma TAC did not change significantly in response to DW either before or after dietary oat supplementation, however dietary supplementation regimen resulted in a significant increase in TAC regardless of exercise status or dietary AVA concentration (*P* < 0.05; Figure 6).

Erythrocyte SOD activity was unchanged with DW before the dietary supplementation regimen (Table 2). Following supplementation, SOD activity was significantly greater in high AVA compared to Control group 48 h after DW (*P* < 0.05, interaction). Erythrocyte GPx activity was not altered by DW but showed a trend toward a lower level in high-AVA vs. Control at Rest (P < 0.1, interaction).

**Table 2:** Erythrocyte antioxidant enzyme activity and plasma glutathione status

		**SOD**		**GPX**		**GSH**		**GSSG**		**GSH:GSSG**	
		**C**	**AVA**	**C**	**AVA**	**C**	**AVA**	**C**	**AVA**	**C**	**AVA**
Pre-Supplementation	Rest	408 ± 20.6	410 ± 19.9	440 ± 18.9	452 ± 16.2	5.90 ± 0.19	5.90 ± 0.10	1.10 ± 0.09	1.10 ± 0.13	5.36 ± 0.20	5.36 ± 0.19
	24 h Post-DW	393 ± 34.6	436 ± 10.0	399 ± 19.7	445 ± 19.3	6.57 ± 0.19	6.26 ± 0.12	1.15 ± 0.08	1.20 ± 0.10*	5.71 ± 0.21	5.20 ± 0.04
	48 h Post-DW	383 ± 15.3	403 ± 20.3	406 ± 14.1	436 ± 50.2	6.44 ± 0.21	7.05 ± 0.12	1.07 ± 0.10	1.14 ± 0.13	6.03 ± 0.22*	6.18 ± 0.17*
Post-Supplementation	Rest	374 ± 38.7	419 ± 21.1	419 ± 14.1	319 ± 17.9¥	5.76 ± 0.17	6.46 ± 0.20	1.07 ± 0.07	1.16 ± 0.12	5.41 ± 0.15	5.59 ± 0.13
	24 h Post-DW	353 ± 23.6	362 ± 60.1	376 ± 11.9	312 ± 24.6¥	6.00 ± 0.15	6.65 ± 0.18	1.19 ± 0.05*	1.19 ± 0.13	5.03 ± 0.17	5.57 ± 0.10
	48 h Post-DW	389 ± 17.5	449 ± 6.5§	397 ± 11.9	336 ± 61.9	6.27 ± 0.08	6.48 ± 0.15	1.02 ± 0.07	1.10 ± 0.06	6.16 ± 0.16*	5.89 ± 0.17

Note: Data are mean ± SEM (N = 8). SOD, superoxidae dismutase. Gpx, glutathione peroxidase. GSH, reduced glutathione; GSSG, glutathione disulfide. DW, downhill walking (see text for details). * P < 0.05, 24 or 48 h Post-DW vs. Rest. § P < 0.05, Post-supplementation vs. Presupplementation. ¥ 0.05 < P < 0.1, AVA vs. Control.

Plasma GSH concentration was not significantly altered by DW or dietary supplementation (Table 2). Plasma GSSG concentration increased significantly (*P* < 0.05) 24 h after DW and returned to baseline levels at 48 h both before and after supplementation. Change in GSSG was not affected by AVA content in the diet. Plasma GSH:GSSG ratio did not change at 24 h but was significantly increased 48 h post-DW vs. Rest both before and after dietary supplementation (*P* < 0.05). No difference between AVA and Control groups was observed.

## Discussion

In the present study, DW resulted in a significant, though modest, increase in CK activity after 24 h indicating that the DW protocol was sufficient to elicit muscle damage among older womon subjects. The repeated eccentric contractions of DW might have caused sarcomere stretching and membrane damage, leading to the escape of sarcoplasmic constituents such as CK to the circulation. AVA supplementation did not affect the extent of muscle damage due to DW, as the CK response was unchanged after supplementation.

Previous studies have shown that a single bout of unaccustomed eccentric exercise can lead to a protective effect whereupon a second bout of eccentric exercise may result in less muscle damage [32-34]. This effect was not observed in this study possibly because the 8 week period between DW sessions was a sufficiently long washout period and the DW protocol was mild providing relatively small stimulus to the muscles involved. Furthermore, postmenopausal women lack estrogen, which may provide membrane stability by intercalating among plasma membrane phospholipids. Therefore, the skeletal muscle of postmenopausal women may be more prone to DW-induced damage compared to younger subjects [13].

### AVA supplementation reduced plasma inflammatory markers

The local response to eccentric contraction-induced muscle damage is the triggering of inflammation [35]. A major part of the muscle repair process is the arrival and infiltration of neutrophils at the site of damage followed by phagocytosis. During this process, NADPH oxidase activity increases in a respiratory burst to convert O_2_ to superoxide radical and cause oxidative damage [36]. In the current study we did not obtain muscle biopsy samples to test this scenario, however, concomitant with the increase in CK 24 h post-DW, NRB increased significantly in circulating neutrophils (Figure 2). This finding suggests that DW-associated muscle damage might have stimulated either receptor binding or NADPH oxidase activity, or both of the circulating neutrophils.

After 8 weeks of dietary oat supplementation, DW triggered a NRB level at 24 h in the Control group, similar to the response seen prior to the dietary regimen. However, high-AVA supplementation resulted in a protection against this response. In a previous study, Brickson et al. [37] showed in a rabbit muscle stretch injury model that M1/70 antibody could block the iC3b domain of neutrophil receptors for respiratory burst without affecting its adhesive effect. Previous work conducted in HAEC demonstrated that AVA was able to inhibit both adhesion and inflammatory cytokine productions [38]. Our data were the first time to show that AVA could suppress neutrophil respiratory burst activity in human in response to physical stress and thus support the notion that AVA is a potent anti-inflammatory agent.

A key step for the signal transduction of mononuclear cells is NFκB activation [39,40]. Peripheral blood mononuclear cells isolated from patients with certain inflammatory diseases and pathogenesis showed increased NFκB binding. Because previous studies showed that AVA could block NFκB signaling in vitro [38,41], we measured NFκB binding to DNA in the nuclear extracts of mononuclear cells ex vivo. We found that older women receiving 8 weeks of high-AVA diet showed significantly reduced NFκB binding in mononuclear cells compared to those who received low-AVA control diet both at rest and during the two day post-DW recovery period (Figure 5). These effects may be of particular importance in postmenopausal women, who suffer from increased risk of inflammation.

DW appeared to elicit a whole-body inflammatory response among the older women as indicated by elevated CRP levels in the plasma 48 h post-DW, a reliable marker of systemic inflammation [42] Furthermore, resting CRP levels in older women were twice as high compared to a study we conducted with young women at 18–25 year of age [43]. However, 8 weeks of high-AVA supplementation completely abolished CRP response to DW seen in the control diet group at 48 h. Inflammation is a double-edged sword. While at young age inflammation in response to heavy muscle contraction especially eccentric contraction is viewed as a necessary process for muscle to recover from injury, among aged human subjects muscle growth and repair appear to be inhibited at high level of inflammation [11]. Furthermore, systemic inflammation has been shown to disrupt local inflammatory responses that are responsible for muscle repair [35]. The repercussions of a lifetime of repeated inflammatory response are thought to be experienced in the aged individuals as the result of a process known as “inflammaging” [6].

### AVA supplementation suppressed pro- inflammatory cytokine production

Inflammation is associated with increased pro-inflammatory cytokine production, which could occur in both activated leukocytes and injured muscle cells [35]. In the current study, DW did not significantly increase plasma levels of three major pro-inflammatory cytokines among older women, presumably because the DW protocol was not strenuous enough. However, plasma IL-1β concentration was decreased at rest and 24 h post-DW among subjects receiving high-AVA supplementation, whereas TNF-α level showed a trend of reduction. It is known that plasma IL-1β is associated with increased adhesion molecule expression, hypothalamic modulation of body temperature, and hyperalgesia [44]. By reducing IL-1β, AVA might have decreased leukocyte invasion and, together with the decrease in neutrophil respiratory burst, protect skeletal muscle from elevated inflammatory status seen among older women [45]. Reduced plasma TNF-α might also play a role in attenuating NFκB activation and NRB in response to DW. Guo et.al [38] reported that the inhibitory effect of AVA on the expression of pro-inflammatory cytokines was mediated through NFκB activation in HAEC. Our finding that AVA could suppress NFκB activation in human plasma mononuclear cells provided further evidence that AVA may inhibit inflammatory responses through a common pathway of NFκB.

### AVA had no adverse effect on blood antioxidant and redox status

Besides AVA, oats contain a variety of phytochemicals such as phytic acid, polyphenols, flavonoids, and tocols that function as antioxidants [18]. Thus, oat supplementation, regardless of AVA concentration, significantly increased plasma TAC indicating an overall antioxidant protection. There has been a concern, however, about a high dose of dietary antioxidant supplementation that may cause some adverse effects such as interference with intrinsic antioxidant systems and preventing exercise-induced metabolic benefit [17]. In older women receiving 8-weeks of high-AVA supplementation, erythrocyte SOD activity showed no change, whereas GPx activity showed a modest but significant reduction (Table 2). Plasma GSH content and GSH:GSSG ratio were normal both at rest and in response to DW. These data indicate that despite the clear benefit of anti-inflammatory effects, high-AVA supplementation did not cause major adverse effects on the endogenous antioxidant system. The modest drop in GPx activity in red blood cells should not raise a concern as the GPx in vivo activity has a large margin of protection due to its relatively low Km (~1 mM).

## Conclusions

High levels of dietary AVA significantly decreased systemic inflammatory response of the older women to downhill walking as indicated by lowered neutrophil respiratory burst activity and plasma CRP concentration. AVA supplementation attenuated plasma IL-1β levels and suppressed mononuclear cell NFκB activation. These effects did not adversely affect the endogenous antioxidant system. Thus, dietary supplementation of AVA at the given dose appeared to be a useful dietary supplement in reducing inflammation after demanding physical exercise.

## Abbreviations

AVA: avenanthramides; BMI: body mass index; CK: ceatine kinase; CRP: C-reactive protein; DW: downhill walking; GSH: glutathione; GSSG: glutathione disulfide; GPx: glutathione peroxidase; IL: interleukin; (NF) κB: nuclear factor-kappaB; NRB: neutrophil respiratory burst; ROS: reactive oxygen species; SOD: superoxide dismutase; TAC: total antioxidant capacity; TNF-α: tumor necrosis factor-α.

## Competing interests

The authors declare that they have no competing interests.

## Authors’ contributions

RK and LLJ designed research; RK, JRD and CK conducted research; RK analyzed data; RK, CK, YC, and LLJ wrote the paper; TOZ formatted the paper; LLJ had primary responsibility for final content. All authors read and approved the final manuscript.
